# Our Skin, and How to Take Care of It
**The Management of the Skin and Hair*. Malcolm Morris, F.R.C.S.E.—Cassell & Co., London, Melbourne, and New York.


**Published:** 1887-03-05

**Authors:** 


### Our Skin, and How to Take Care of It.*

THE area of skin in a man of average size is equal to about twelve square feet—in a tall man it may be eighteen square feet. So says the learned Sappey. As man thus carries about with him skin sufficient to make a respectable hearthrug, or to cover an indefinite number of cricket-balls, one would think he would take some pains to understand its function and to treat it wisely. But not he. From day to day he rubs his face, and from week to week he scrubs his body, and thinks no more about it. He rubs and scrubs because his fellows do it, otherwise, he would not. Does not Dr. Livingstone tell us of an old African chief who admitted he had once washed, but that it was so long ago, he could not remember what it was like! Does not the same writer also tell how he got rid of an importunate native by threatening to take him down to the river and wash him. No; man in his primitive condition cannot be called a washing animal. It is only when he rises high

* *The Management of the Skin and Hair.* Malcolm Morris, F.R.C.S.E.—Cassell & Co., London, Melbourne, and New York.

in the scale of intelligence that he appreciates the virtues of soap and water; and great is the virtue of the soap and water bath; it cleanses, not merely the outward man, but penetrates even to his moral nature. Who does not feel more charitably disposed towards his fellow-men after having undergone the invigorating and cleansing process of the Turkish Bath? One's petty sins and miseries are for the present sweated out; and one leaves with a cleaner moral as well as material covering.

Science is no respecter of mysteries; it pokes its nose into all places, even the most sacred. Mr. Morris is the scientific Asmodeus of the Toilet Temple; he lifts the roof and reveals to the gaze of the astonished multitude all its mysteries, from the rouge-pot to the hair-dye. Not only reveals them, but analyses and dissects them, till there is no mystery left. For hundreds of years has man daily rubbed soap upon his chin to simplify the always troublesome process of shaving. He knows it would be about as difficult to make good bricks without straw as to shave well without soap. How the straw acts, man might, by taking much council with himself, guess; but we doubt if one out of a hundred could tell how the soap acts on the hairy stubble, for it is not by softening it, as is generally supposed. Yet there is a simple explanation of it—as there is of most things—which explanation we leave our readers to puzzle over, or to find out by reading Mr. Morris's little book. Few books of Messrs. Cassell's Health Series will be read with greater interest by the public than this. The subject is one which all can understand, and in which all are interested, and is treated in a most skilful manner, Mr. Morris having incorporated with the more practical matter much very interesting historical information. The astonished reader will find that his simple daily toilet is founded upon many sciences; that in the mere washing of his hands with soap and water, he performs a complicated chemical action. He will feel somewhat as Monsieur Jourdain did on finding he had spoken prose for forty years without being aware of it.

